# The Dutch Techcentre for Life Sciences: Enabling data-intensive life science research in the Netherlands

**DOI:** 10.12688/f1000research.6009.2

**Published:** 2016-01-06

**Authors:** Lars Eijssen, Chris Evelo, Ruben Kok, Barend Mons, Rob Hooft, and the other founding members of DTL Data (see Acknowledgements)

**Affiliations:** 1 Department of Bioinformatics - BiGCaT, Maastricht University, 6229 ER Maastricht, Netherlands; 2 Dutch Techcentre for Life Sciences (Foundation office), Catharijnesingel 54, 3511 GC Utrecht, Netherlands; 3 Netherlands eScience Center, Science Park 140, 1098 XG Amsterdam, Netherlands; 4 Leiden University Medical Center, Albinusdreef 2, 2333 ZA, Leiden, Netherlands

**Keywords:** Data, technologies, initiative

## Abstract

We describe the Data programme of the Dutch Techcentre for Life Sciences (DTL, www.dtls.nl). DTL is a new national organisation in scientific research that facilitates life scientists with technologies and technological expertise in an era where new projects often are data-intensive, multi-disciplinary, and multi-site. It is run as a lean not-for-profit organisation with research organisations (both academic and industrial) as paying members. The small staff of the organisation undertakes a variety of tasks that are necessary to perform or support modern academic research, but that are not easily undertaken in a purely academic setting. DTL Data takes care of such tasks related to data stewardship, facilitating exchange of knowledge and expertise, and brokering access to e-infrastructure. DTL also represents the Netherlands in ELIXIR, the European infrastructure for life science data. The organisation is still being fine-tuned and this will continue over time, as it is crucial for this kind of organisation to adapt to a constantly changing environment. However, already being underway for several years, our experiences can benefit researchers in other fields or other countries setting up similar initiatives.

## Introduction

In this introduction we will explain the origin of the Dutch Techcentre for Life Sciences (DTL) and its three programmes Data, Technologies and Learning. Furthermore, we discuss how the activities of the DTL Data programme fit in the parallel development of data stewardship and knowledge structuring initiatives in science overall.

### Why was DTL started?

The initiative for DTL was based on the growing complexity of life sciences projects requiring multidisciplinary collaboration, coinciding with an increase in variety and volume of data. Starting 2003 several technology specific institutes developing technical services and techniques for life sciences have been set up in the Netherlands and operated until 2013. These include the Netherlands Bioinformatics Centre (NBIC), Netherlands Metabolomics Centre (NMC), Netherlands Proteomics Centre (NPC), and the Netherlands Consortium for Systems Biology (NCSB). These institutes were put in place to foster technology research, drive the exchange of methodology among labs and translate these into technical services that other scientists could use. Around 2012 it became clear that in the future the development and use of these technologies would no longer receive similar direct funding, and that research projects that apply a technology would need to budget for that. Coinciding with the increasing need for support of multidisciplinary collaboration and research projects, the technology institutes decided to develop a framework in which they would continue to exchange expertise across technology disciplines, build up a collective and well-accessible research infrastructure (RI) and deliver the services required. This has led to the formation of DTL.

The major advantages of this form of organisation are:

- Technology programmes working together in a single organisation enable the application of what we call *integrated life science research* requiring the use of multiple technologies in a single research project, and the integration of generated and already available data.
- Members of DTL can collaboratively draw attention to the fact that the fundamental developments in the technology fields require more attention of both the collaborating research organisations as well as the national funding agencies. Together we can look for solutions to tackle these challenges.
- Establishing a collective technology platform of the major research organisations in the Netherlands provides further chances to establish international partnerships for individual member organisations or as a collective.

An important distinction between DTL and other institutes with similar functions in other countries is that DTL was not set up as an institute by a (national) funding organisation (like e.g., the National Centers for Biomedical Computing in the US ^1^, and NECTAR in Australia, http://hdl.handle.net/1885/8993), but as a collaboration institute funded primarily by partner organisations. Where such bottom-up efforts to set up a supporting organisation are seen, they are often localised to a single research institute ^2^, and rarely started as a public-private partnership.

### Timeline

During the preparatory phase of ELIXIR ( elixir-europe.org), in 2012, several high profile bioinformatics and systems biology representatives started an initiative called DISC, The *Data Integration and Stewardship Centre*. They met several times to discuss the implementation of ELIXIR in the Netherlands. In parallel to this, the initiative to establish the Dutch Techcentre for Life Sciences was launched on the 31st of October 2012. The DTL organisation was started as a platform of leading universities, research institutes, university medical centres, science funders, government funding sectors (‘topsectoren’ in the Netherlands) and private companies from the health, nutrition, agrigenomics and industrial microbiology and information engineering sectors. We soon discovered that there was a significant overlap in the goals of the two initiatives, and it was decided to merge DISC into DTL as its Data programme. Starting from the 1st of January 2014, organisations have been signing up for formal membership of DTL.

### DTL programme areas

As mentioned above, DTL has organised its actions in three areas, Data, Technologies and Learning, which run as individual but cross-connected programmes within the organisation.

*DTL Learning*, to start with the third area, manages an inventory of all training needs and offerings in life science technologies. It forms the bridge to the national Research School on Bioinformatics and Systems Biology (BioSB, biosb.nl) and other related *research schools*, and maintains contacts with all academic institutes that offer bioinformatics bachelor and master programs or postdoctoral training. DTL Learning also bundles expertise available in the DTL network, and organises both *ad hoc* and repeated training and courses on diverse subjects related to developments in the Data and Technologies programmes.

*DTL Technologies* bundles more than 100 research labs that offer support to life scientists with different technologies (so called *technology hotels*). These technology hotels include a wide coverage of a variety of experimental (e.g. next generation sequencing, proteomics, metabolomics, bioimaging) technologies as well as bioinformatics and systems biology expertise. DTL Technologies facilitates the contact between the technology hotels and external researchers as potential customers *e.g.* through the organisation of funding calls that encourage new collaborative projects. In the DTL Technologies programme we will also work on harmonising and optimising access to hotels to make it easier for life scientists to use the latest technological opportunities and access multiple facilities in parallel.

*DTL Data* brings together experts on every aspect of data stewardship, tools and databases, and e-infrastructure. DTL Data builds relations for the people involved in the other DTL programmes and partner organisations and connects to international initiatives such as ELIXIR, the pan-European life science research data infrastructure. The setup of DTL Data has gone hand in hand with more generic developments related to data and knowledge handling in the life sciences that we will address below, before describing the DTL Data programme in more detail.

**Figure 1.**
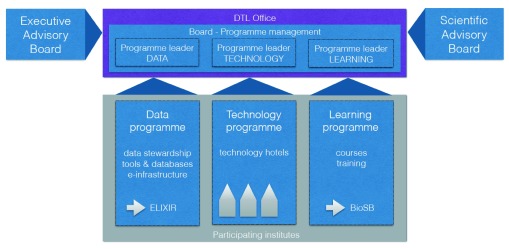
Organisation of DTL and its programmes. The board of DTL is advised by executive and scientific committees. For each of the programmes, Data, Technology, and Learning, it contains a programme leader who coordinates the work done by the DTL partners in the programme and takes care of the coherence with the other two programmes.

Whereas all scientific work in each of the three programmes is carried out within the participating institutes, DTL is run as a small not-for-profit organisation that has a central operational team governed by a board of domain representatives. The team takes care of organisational tasks to support and manage the functioning of the three programmes. The coherence between the three programmes is guaranteed by the participation of the three programme leaders. Operations are monitored by a scientific advisory committee consisting of senior staff from academia and industry and the board is advised by executive representatives from the partner institutes ( Figure 1).

### Parallel developments: data stewardship and knowledge structuring

Driven by the rise and wide application of modern data-intensive technological approaches, present-day research projects in the life sciences collect much data that intrinsically has more value than the project itself will extract. This valuable and costly to obtain data offers many opportunities for re-use, which makes serious investment in keeping the data available and properly annotated (data stewardship) a more effective strategy than reacquiring the data. This also opens up the possibilities for research to reach new conclusions based on existing data. Furthermore, good data stewardship is required to make the work reproducible. Initially in the US and later in Europe, funding agencies have started demanding data stewardship to be an integral part of all scientific research projects. Besides data stewardship, integrative research requires proper structuring of knowledge based on aggregated and possibly curated findings of previous research.

DTL facilitates data stewardship and knowledge structuring in all associated projects through participation in the development and deployment of an initiative to make data Findable, Accessible, Interoperable, and Reusable (FAIR, datafairport.org). Making data and knowledge sources findable and accessible by both humans and computer systems requires a standardised description of metadata and study capturing as well as long-term storage and proper licensing. Interoperability and reusability require the representation of data and knowledge in such a way that they can be easily combined and used for further analytical processing ^3^.

To support practical implementation of good data stewardship, DTL and its Data programme are on a mission to bring together all experts that can help life scientists with different aspects of their data management, and to show life scientists that it is not efficient to do everything in house using local solutions.

The remainder of this paper describes the organisational structure and approaches of the DTL Data programme in more detail.

## Content of the DTL Data programme

DTL-associated scientists and engineers are responsible for data integration and stewardship in various life science initiatives in different life science sectors. They bring in expertise, reusable tools and databases that have been developed in the Netherlands or elsewhere, and have access to a shared e-infrastructure.

### Bioinformatics and medical informatics expertise

DTL brings together experts with a very diverse professional expertise in life science data management. This expertise is classified along four independent dimensions:

- The life science *sector*: current activities are in health, agri/food, nutrition, and industrial biotechnology.
- *Location*: even though the Netherlands is a relatively small country, a local expert is sometimes preferred for an advice or in a collaboration.
- *Phase* in the data lifecycle: we distinguish expertise in planning an experiment, collecting data, data processing, data analysis, data and knowledge integration, and modelling. There is also underlying expertise in biostatistics, systems biology, instrumentation, data security, computing infrastructure, and computer science approaches.
- Technical *discipline* and type of data: *e.g.* genomics, proteomics, metabolomics, bioimaging, biobanking, knowledge representation.

To make expertise fulfilling requirements on all four dimensions available to life scientists all over the country, we are working on setting up a network of local expert centres at different sites. Such expert centres can function as help desks: places where information can be obtained about the expertise available locally as well as elsewhere. Representatives of the expert centres are in frequent contact with each other to learn about new developments and learn of each other’s experience (both in techniques and in organisation). Over time, DTL will also extend its own help desk that can guide people to the right expert centres.

A very important mission of DTL Data is to prevent projects from running into problems because of *unconscious incompetence* ^4^; we want to facilitate early interaction between life scientists with a specific plan and experts in all the technical fields that they need to engage, to avoid underestimating technological tasks or risks.

### Tools & databases

Many researchers and other experts have (co)developed reusable tools and databases. They have ample experience to implement these in different projects. Such tools can often be implemented in a new project using an existing deployment with dedicated user support. In other cases, specialised installations of the software can be made, tailored to the project. DTL has a strong preference for reuse of existing tools, which have proven their value in earlier national or international projects. Advantages of such tools are that they have overcome their teething problems, that their continued development benefits multiple projects, and that the reuse increases interoperability with other tools and existing data.

DTL, at this moment, is not performing any selection or endorsement of tools and databases. DTL Tools are supported by DTL partners that have developed the tools or by power users in the organisation. DTL, however, is actively involved in the inventory of Dutch tools for the ELIXIR registry ( https://bio.tools/). In future, DTL may suggest selected unique, scalable, and internationally supported tools to be accepted for inclusion in the ELIXIR programme.

### e-Infrastructure

In the past, many life science labs have each been taking care of their own needs for computing. More and more, however, the data to be processed becomes too large to handle. Furthermore, server system maintenance is not a core competence of a life scientist, and keeping a local cluster running should not be the task of a PhD candidate. Computing and data storage are becoming an infrastructure: equipment that nobody can do without, and which is inefficient to duplicate for every project. Many groups are therefore no longer willing to maintain the needed infrastructures themselves, and set up institutional services together employing specialised people for maintaining the computing equipment. Additional benefits of such centralisation efforts are flattening-off peak demands and allowing individual projects to be run at relatively short notice. Also, it reduces the need for synchronising purchase of new equipment with the start of new projects, which without central facilities results in waste for short projects and the use of outdated computing resources for longer projects. DTL brings experience from centralisation efforts together, and ensures alignment with the national centres for computing as well as international e-infrastructure projects like the European Grid Initiative (EGI, www.egi.eu) and EUDAT ( www.eudat.eu). DTL links to and between the people that work on harmonising the computer centres so that migration of computing work and federation of resources become easier. When a new data intensive life science project is started with new demands for computing or storage, the best solution for the location of such computing is found in collaboration.

The e-infrastructure that can be shared is not limited to the computer racks (Infrastructure as a Service, IaaS). We also investigate possibilities for sharing higher level platforms (Platforms as a Service, PaaS), for example the workflow supporting software Galaxy ^5^, which has been supported by the Netherlands bioinformatics centre in the past, and potentially other shared infrastructures for systems biology. We are also working together on a shared data publishing infrastructure based on experience from the Open PHACTS project ^6^.

## Organisation of the DTL Data programme

### Organisational structure and facilitation

**Figure 2.**
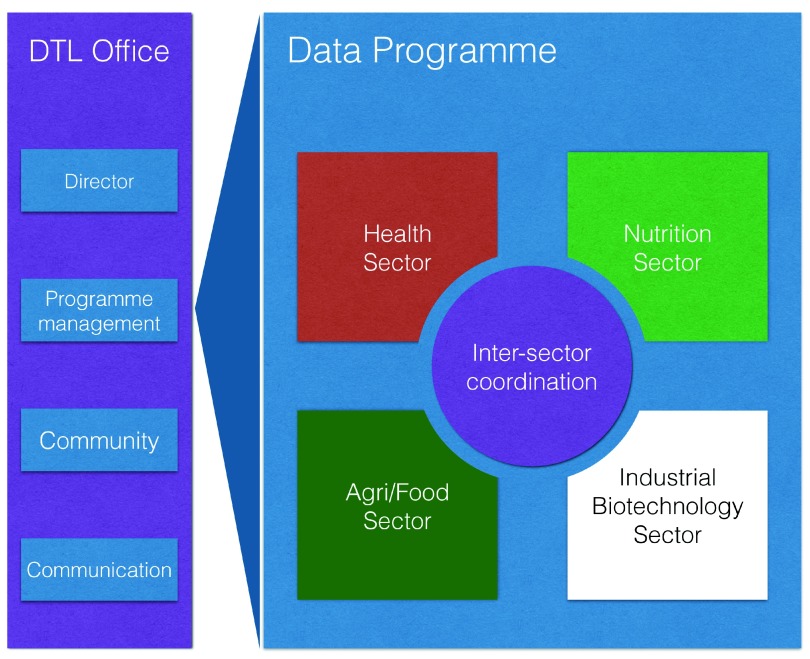
Organisation of DTL Data. DTL has a small office that organises and coordinates programme management, community and communication for the different programmes. The actual DTL Data projects are run by the partners outside of the DTL Office. The coordination structure is primarily divided into four sectors.

The DTL Data programme is coordinated by a programme manager from the DTL Team. All projects are executed by DTL partners outside of the team. The primary organisation of DTL Data is per sector of life science research ( Figure 2). We organise several kinds of meetings for different target groups, which we have identified as fulfilling an urgent need: project leader meetings, programmer meetings and so-called *focus meetings*. We also identify people with similar interest and facilitate interest groups and working groups with their own meetings. Each of these types of events will be described in more detail.

***Project leader meetings***

Parallel running scientific projects often share common needs, which could be addressed by common efforts. Also, projects can often benefit from previous experience by others that are not directly involved. To detect such synergies, DTL organises project leader meetings for each life science sector in which project representatives meet regularly to discuss their progress and intentions. For the healthcare sector, this is already in place; the other sectors (agrigenomics, nutrigenomics and industrial biotechnology) are now setting up similar meetings. The principal project leaders of the four sectors will be meeting together on a monthly basis to discuss progress and to identify synergies between the sectors.

***Programmers meetings***

Many of the programmers involved in the bioinformatics projects in the different sectors of DTL Data are so-called *embedded programmers,* often the only bioinformatician in a biology or medical setting. Others work together in groups. In order to keep each other informed and to encourage interactions between these programmers, DTL Data calls them together every two months for lectures and workshops on topics ranging from programming techniques to biological applications.

***Focus meetings***

During our work we regularly recognise similar problems or solutions being raised in more than one context. These common interests can be signalled by the programme managers or brought up by DTL scientists. For such topics we organise *focus meetings*. A focus meeting brings together a group of people that preferably have never met in that composition, to discuss a subject that is either crossing borders between technologies or between sectors. Focus meetings are not only organised by DTL Data, but also by the DTL Technologies and DTL Learning programmes. A focus meeting often contains a few short lectures, followed by a well-prepared discussion that engages the whole audience. After the meeting, a white paper is written by the organisers of the meeting that is published on the DTL website.

***Interest and working groups***

If a group of people, *e.g.* after a focus meeting, feels the need to exchange experience more often, they can form a so-called *interest group* within DTL. DTL facilitates these interest groups with meeting rooms, and tries to find a young researcher as a champion of the group to keep it going. This is modelled after “Project and Area Liaisons” (PALs) from earlier EU and UK projects ^7^. PALs are rewarded for introducing new ways of working: they are provided with extra support for their work and direct influence on the development of the new working methods.

An interest group that has identified an issue they want to work on together can form a *working group*. A working group needs to be supported by a part-time project leader to take the practical work out of the hands of the principal investigators. Each working group must deliver a practical result (deliverable) after a limited time. DTL is looking for ways to support the working groups by providing resources for the project leaders.

Both interest groups and working groups can be supported with a good software development environment, mailing lists, a website and a wiki to exchange information.

### Relations with other DTL programmes

The Data programme interacts with many organisations, both internal to DTL (other programmes and partners) as well as external. We first describe the internal interactions.

***Help desk, training and education***

In the day to day operations of the Data programme, we frequently come across needs for training: both training for data scientists to broaden their knowledge with newly developed technologies, as well as training for life scientists to make them aware of and teach them how to use solutions that are being developed in DTL Data projects. This is expected to become even more important once the development of local data desks in different institutions will be realised. The setup of these data desks will bring together experienced data scientists from different institutes, and they will find out that others have complementary expertise that they sometimes need to replicate. Also, life scientists with less experience will have a low barrier to approach their local data desk for advice, bringing in more demand for basic data awareness training. All of these training needs will be developed with the DTL training Programme, which is very well connected to people and organisations that can support this effort.

***Data-related technology hotels***

Many of the people involved in DTL Data offer their services to life scientists as a Data hotel in the DTL Technologies Programme. DTL Data works with DTL technologies to define the needs of and requirements for these data-specific hotels. An overview of current DTL hotels is available at www.dtls.nl/expertise-services/hotels.

### Relations with external programmes

External connection of DTL Data include for instance projects from the Innovative Medicine Initiative (IMI, www.imi.europa.eu) and infrastructures under the European Strategy Forum on Research Infrastructures (ESFRI) scheme, including ELIXIR.

***ELIXIR***

Synchronous with the development of the DTL organisation, bioinformatics institutes and laboratories all over Europe have set up the European research infrastructure for life science data and bioinformatics, ELIXIR. ELIXIR is organised as a hub hosted at the EBI in Hinxton, UK, and nodes in each of the member countries. In the Netherlands, DTL hosts the ELIXIR node (ELIXIR-NL). Each of the nodes contributes specific expertise to the ELIXIR network. The Dutch node contributes expertise related to interoperability, learning, and computer and network infrastructure. Through the network, DTL-associated scientists can benefit from all European contributions.

DTL and ELIXIR have developed the concept of so-called *Bring Your Own Data* (BYOD) meetings as a platform to bring together data owners and data experts. Also biological domain experts are invited where relevant. The main goal of these meetings is to get data owners acquainted with the possibilities to connect and functionally interlink their data with other datasets and knowledge resources by applying FAIR principles. Researchers can suggest a BYOD party and DTL will assist with the logistics and invite data experts.

***Other ESFRI programmes and national projects***

Europe has many other Research Infrastructures in the life sciences, each with their own special focus. Also in the Netherlands several larger project organisations are active in life science research. All of these have their own research data and associated challenges. In the Netherlands we make sure that the people working with that data are co-developing and steering the DTL Data Programme. This ensures that the methods and tools they use are compatible with the ELIXIR choices and avoids unnecessary duplication of development efforts.

## Conclusion

Life science research becomes more and more data intensive and cross-disciplinary at unprecedented scales. Individual research groups do not have the resources and the interest to keep in contact with all expert providers and keep informed of the progress of other related projects at such scales. In the Netherlands we have developed a networked approach to accommodate for the challenges posed by modern data-intensive life science research. The establishment of DTL as a collective platform that brings together experts in various technological disciplines across life science domains, facilitated by a small core team, allows projects to run efficiently. Already in the preparatory period and in the first year of operations we have identified synergies between parallel running research projects and found common interests across researchers with a focus in surprisingly different disciplines. The growing community of experts involved in DTL Data makes sure that necessary data-related expertise can be located for any researcher in the life sciences starting on any new project. At the publication date of this article DTL had over twenty confirmed member organisations. The current partner list can be found at www.dtls.nl/about/partnership/.

### Contact

To find out how DTL Data can support your challenges or for more inquiries about the setup of the organisation, contact Rob Hooft (programme leader) at rob.hooft@dtls.nl

For further information on the other programmes of DTL contact Ruben Kok (director DTL) at ruben.kok@dtls.nl

Website: www.dtls.nl
