# Supplementary material for: The Dutch Techcentre for Life Sciences: Enabling data-intensive life science research in the Netherlands
Source: F1000Res. 2016 Jan 6;4:33. Originally published 2015 Feb 2. [Version 2] doi: 10.12688/f1000research.6009.2 (PMC4743138; doi:10.12688/f1000research.6009.2)
Supplement: Peer review history

# Peer review: Referee response for version 2

**DOI:** 10.5256/f1000research.8266.r11903

**Published:** 2016-02-09

**Authors:** Steffi Suhr

**Affiliations:** 1 BioMedBridges, European Bioinformatics Institute, Hinxton, UK

The authors have done a substantial amount of work to restructure, clarify and refocus the manuscript. I am happy to approve this new version.

I have read this submission. I believe that I have an appropriate level of expertise to confirm that it is of an acceptable scientific standard.

# Peer review: Referee response for version 2

**DOI:** 10.5256/f1000research.8266.r11902

**Published:** 2016-02-02

**Authors:** Karin Verspoor

**Affiliations:** 1 Department of Computing and Information Systems, The University of Melbourne, Melbourne, VIC, Australia

I thank the authors for their excellent work in overhauling this manuscript. I find it much improved in terms of clarity and structure. The paper does a nice job of introducing the Dutch Techcentre for Life Sciences, and describing an alternative model to providing ongoing support for modern data-oriented life sciences research.

I have read this submission. I believe that I have an appropriate level of expertise to confirm that it is of an acceptable scientific standard.

# Peer review: Referee response for version 1

**DOI:** 10.5256/f1000research.6430.r7550

**Published:** 2015-03-24

**Authors:** Bryn Williams-Jones

**Affiliations:** 1 ConnectedDiscovery, London, UK

This article outlines the background, formation and to an extent the operation of the Dutch Techcentre for Life Sciences (DTL) with a focus on the data programme, DTL-Data. Given this type of organisation is of relevance to data researchers in other nations, researchers in other fields, and set against a background of increasing data complexity and institutional networking in the wider community, this could provide a model for other inititatives. 

In common with the other reviewers, there are some issues with the presentation which would help readers new to DTL to understand the background, structure and operation of this entity. To encourage similar organisations in other nations, the historical background is relevant, but could be split to a separate section - as a text box/diagram or similar to aid the flow of the description of DTL operations as they are now.

Whilst DTL-Data is discussed in some detail, the other sections are briefly mentioned and its challenging to gain a picture of the holistic DTL and the competencies necessary to establish a similar organisation in other nations. Again, as noted by the other reviewers, an outlook comparing other national efforts, their relation to DTL, what may have particularly aided development of DTL in the Netherlands from both a structural/funding and cultural/operational perspective would be useful. Similarly examples of FAIR data principles, information on some of the tools and databases mentioned but not named could help researchers in other nations contextualise the assertions made. This may be of more value to those less familiar with the operations of DTL than detailed description of the meetings which would be better summarised in less detail.

I have read this submission. I believe that I have an appropriate level of expertise to confirm that it is of an acceptable scientific standard, however I have significant reservations, as outlined above.

# Response

**Published:** 2015-12-29

**Authors:** Lars Eijssen

**Affiliations:** Maastricht University, Netherlands

Thank you for your feedback. Herewith our responses to the points raised.

Response to "In common with...as they are now":

According to the suggestion, we have added a separate ‘timeline’ box to describe the historical context of DTL. Furthermore we have made more clear right from the opening of the paper that it is mainly about the Data programme, and described the remaining DTL context more in connection to this focus.

Response to "Whilst DTL-Data...in other nations":

Even when trying to keep the text on the other parts of DTL brief, and making more clear in the text that this paper specifically addresses the DTL Data programme, we have added an extra short paragraph and a diagrammatic figure  (Figure 1 and the paragraph directly preceding it) on the organisation of DTL as a whole.

Response to "Again, as noted by...summarised in less detail":

We rephrased the ‘Why was DTL started’ subsection to more clearly introduce its benefits and reasons for setup and to extend the description of the projects that were running in the Netherlands before DTL, to better sketch the national operational and funding perspectives. We have substantially reduced the level of detail in our description of the meetings. For (stewardship and other) tools and databases, we deliberately had not included examples in order not to select some above others, since DTL partners are the entities that are offering and supporting tools, rather than DTL centrally, which does not enforce specific ones. We now included a paragraph to the manuscript (also in response to a similar point made by the first reviewer) clarifying this aspect and mentioning the relationship between DTL and ELIXIR with respect to tool selection, referring to the latter’s tool registry (‘Tools & databases’ subsection).

# Peer review: Referee response for version 1

**DOI:** 10.5256/f1000research.6430.r7547

**Published:** 2015-03-16

**Authors:** Karin Verspoor

**Affiliations:** 1 Department of Computing and Information Systems, The University of Melbourne, Melbourne, VIC, Australia

This paper describes the structure and role of the Dutch Techcentre for Life Sciences (DTL), with a specific emphasis on the DTL Data programme. The DTL is an important initiative in the Netherlands to support effective and efficient data-intensive life science research throughout the Dutch research community by facilitating the connection of researchers with computational tools, expertise, and infrastructure.

As the first reviewer has identified, there are some structural issues in the presentation of this paper that could improve its focus and clarity. There are also some more minor issues, for instance a lack of background on certain referenced programs when first mentioned (ELIXIR is introduced in the second paragraph of the Introduction, but not defined until several paragraphs later; not all readers may be familiar with the program).

Elaborating on the first reviewer's point about e-infrastructures, it would be helpful for the authors to relate the model described in the paper, as developed for the DTL, to other possible models that exist, for instance the National Centers for Biomedical Computing in the US (see e.g., http://jamia.oxfordjournals.org/content/jaminfo/19/2/151) or bioinformatics core facilities in place at a more local level (see e.g., the discussion at http://bioinformatics.oxfordjournals.org/content/27/10/1345). Given the emphasis on data management and computing infrastructure of the DTL Data programme, cloud-based generic e-research infrastructure supported at a national level (e.g. for social sciences research or the Australian National eResearch Collaboration Tools and Resources (NECTAR), e.g., the Genomics Virtual Laboratory and other Australian research infrastructure programs) are also relevant. I believe that understanding how the DTL Data model is different or unique in the global context is important to support the authors' goals of providing insight to new efforts. Furthermore, it would be helpful to more clearly distinguish the role of the DTL from the national centres for computing to which the DTL is aligned (p. 4).

There are a few minor issues with wording choices that the authors may wish to revisit, e.g. the phrase "the path to professionalisation" -- in what sense is the organisation being "professionalised"? (this recurs in the phrase "professional data stewardship") -- and the phrase "unconscious incompetence" which sounds perhaps more severe than the authors intend.

I have read this submission. I believe that I have an appropriate level of expertise to confirm that it is of an acceptable scientific standard, however I have significant reservations, as outlined above.

# Response

**Published:** 2015-12-29

**Authors:** Lars Eijssen

**Affiliations:** Maastricht University, Netherlands

Thank you for your feedback. Herewith our response.

Response to "As the first reviewer...familiar with the program).":

We improved the introduction of ELIXIR in the text. In the abstract we added context by: “ELIXIR, the European infrastructure for life science data”. Furthermore both in the abstract and the timeline we removed the mentioning of the term ‘ESFRI’, to only introduce this (but now written in full) in the ‘Relation with external programmes’ section by: “…and infrastructures under the European Strategy Forum on Research Infrastructures (ESFRI) scheme, including ELIXIR”.

Response to "Elaborating on ... DTL is aligned (p. 4).":

An important distinction between DTL and other institutes with similar functions in other countries is that DTL was not set up as an institute by a (national) funding organisation (like e.g., the National Centers for Biomedical Computing in the US, and NECTAR in Australia), but as a collaboration institute funded primarily by partner organisations. Where such bottom-up efforts to set up a supporting organisation is seen, they are often localised to a single research institute and rarely started as a public-private partnership. We have also included this text in the manuscript, including suggested references, in the ‘Why was DTL started?’ section.

Related to the last point, DTL has more a role as an orchestrator between research projects, generic computing initiatives, and national computing centres. As suggested by the first reviewer, we added some international initiatives to this phrase, widening the scope. Furthermore, we modified the next phrase (“Together these people work…”) to “DTL links to and between the people that work…” to make DTL’s role as a connector more clear.

Response to "There are a few minor...the authors intend.":

We removed both occurrences of professionalisation/professionalised, as they were superfluous.

The phrase ‘unconscious incompetence’ was a deliberate choice, as a term coined to indicate the issue not being aware of not doing something according to standards. We added a reference to a paper by Kruger *et al.* on this topic.

# Peer review: Referee response for version 1

**DOI:** 10.5256/f1000research.6430.r7548

**Published:** 2015-02-25

**Authors:** Steffi Suhr

**Affiliations:** 1 BioMedBridges, European Bioinformatics Institute, Hinxton, UK

The authors describe the drivers and motivation behind setting up the Dutch Techcentre for Life Sciences and describe the organisational setup so that the experiences gained can benefit researchers in other fields or countries setting up similar initiatives. As the number of initiatives seems to be steadily growing, this is helpful and could indeed provide some useful background against which other efforts could model themselves.

However, trying to come at this from the perspective of someone who might want to implement such an infrastructure, there are some bigger and smaller points that would need to be addressed to be able to give this paper an "approved".

1. The focus is slightly unclear: the abstract and introduction describe the DTL in its entirety, including its history, while more than three quarters are focused on the services related to the last area of activity listed, "DTL Data". The manuscript would benefit hugely from a reorganisation that makes the main focus clearer right at the beginning. It might also be worth considering to put the description of the background and origin (timeline, why was DTL started) at the end of the manuscript. Alternatively, the authors might want to consider leaving the history out of the manuscript completely: at present, the first point that seems to be mentioned is that funding became more scarce, rather than focusing on the benefits.
2. Abstract: as mentioned under (1), the focus on data-related issues should be reflected. It would also be helpful to (very briefly) give examples of "tasks ... that are not easily undertaken in a purely academic setting", as this seems to be one of the core drivers behind DTL. Examples for why and how the environment is changing, and how DTL can address this, would be good in the introduction (rather than leaving this hanging in the abstract).
3. The part about "governance and organisational programmes" is confusing: governance structures are not really described in any detail (who is on the board, and why?). In general, it is unclear that this information adds value to the manuscript - as organisational structures will often heavily depend on local framework conditions, the paragraph on the board and SAB could be left out and the section simply renamed "DTL programme areas" or similar.
4. Section "parallel developments..": the opening paragraph on open data is quite weak and possibly incorrect (e.g. the push towards open data does not originate from the application of data-intensive technologies - arguably, data intensive technologies provide a challenge for open data). For the purpose of this manuscript, less would be more.
5. Going into the Data programme, section on Tools & databases: which tools are supported by DTL? How are they selected and/or prioritised? What is the DTL contribution to these tools?
6. e-infrastructure: It might be interesting to explain if there any interactions with European-wide e-infrastructures.
7. Meetings described: There is an odd level of detail. What is missing is who is involved and why, in particular in the case of the project leader meetings. If these are a continuation of TraiT meetings it is clear that there would be a spirit of collaboration, but what about the "other sectors" mentioned - how were participants identified, how will the aims be stated, how will members be motivated? Focus meetings and working groups: who prioritises the topics, and based on what?
8. Relations with external programmes: ELIXIR - "..gives us the possibility to reach out to experts and tools all over Europe" - how about the other way around, can needs also be identified across DTL that would better be met at the European level?

Minor points (mostly language):

- Page 2: "...and let life scientists each replicate the EXPERTISE at their own institutes, the technology institutes decided to develop a FRAMEWORK in which they..."
- Page 3:
  "..., and Reusable (datafairport.org). ALLOWING data and knowledge sources.."
  "All expertise can be classified along those four dimensions" - delete sentence (redundant). Next sentence, change to: "To make expertise fulfilling all four dimensions available,.."
  "Representatives of the expert centres are in frequent contact.." (delete "involved").
  Change to: "More and more, however, the data to be processed becomes too large to handle."
- Conclusion: "...and found common interests across researchers with a focus in surprisingly different disciplines. ... makes sure NECESSARY data-related expertise.."
- ELIXIR should be listed by its name (without "ESFRI"), with the full name at first mention

I have read this submission. I believe that I have an appropriate level of expertise to confirm that it is of an acceptable scientific standard, however I have significant reservations, as outlined above.

# Response

**Published:** 2015-12-29

**Authors:** Lars Eijssen

**Affiliations:** Maastricht University, Netherlands

Thank you for the extensive and detailed feedback. Herewith a point-by-point response.

1. We rephrased the abstract to directly introduce the Data programme of the DTL, instead of DTL as a whole. We still kept some background on DTL in the introduction, since this gives the context in which DTL Data was set up and lives, but also there we adapted the first lines to mention the programmes, including Data, right away. Furthermore, we put the timeline in a separate box to improve the structure of the text. Also, we rephrased the text to indicate the benefits that clearly underlie both changes in funding policies as well as the Data programme.
2. Data-related issues get more focus in the adapted abstract, as described in the response to the first point. We also added a phrase to exemplify the tasks referred to (“DTL Data takes care of such tasks related to data stewardship, facilitating exchange of knowledge and expertise, and brokering access to e-infrastructure.”). We now also already briefly mention the generic environmental changes right at the opening of the introduction: “The initiative for DTL was based on the growing complexity of life sciences projects requiring multidisciplinary collaboration, coinciding with an increase in variety and volume of data.” For DTL Data specifically, this is further detailed in the ‘Parallel developments: data stewardship and knowledge structuring’ subsection right after the introduction of the three programmes.
3. We have adapted the paragraph in accordance with these comments. We renamed the section and removed the opening line on the board. At the end, we included a short paragraph and a figure on the governance structure, but more focused at its relationship to the programmes including Data.
4. We agree this was not clearly formulated. It is an indirect ‘push’: because of the more generic value such data has, making the data open gets even more important. We rephrased the opening sentence to: “Driven by the rise and wide application of modern data-intensive technological approaches, present-day research projects in the life sciences collect much data that intrinsically has more value than the project itself will extract”. Also, we reformulated the start of the paragraph to indicate that this is the reason for data stewardship (and for funders to ask for this) rather than funder’s requirements being its original cause.
5. DTL, at this moment, is not performing any selection or endorsement of tools and databases. DTL Tools are supported by DTL partners that have developed the tools or by power users in the organisation. DTL, however, is actively involved in the inventory of Dutch tools for the ELIXIR tools registry ( https://bio.tools/). Selection does take place only when the initiative is taken to start supporting the tools or databases in an international context. In such cases the tools are verified to be one of a kind, best of breed and have sufficiently scalable support infrastructure. DTL can then suggest to include the tools in the ELIXIR programme. To be accepted into the ELIXIR programme, tools have to be part of the node plan, which must be accepted by the ELIXIR SAB. No tools have been added to the ELIXIR NL portfolio yet. A shortened version of this text has been included in the ‘Tools & databases’ subsection.
6. To name some important initiatives, we added the clause: “…as well as international e-infrastructure projects like the European Grid Initiative (EGI, www.egi.eu) and EUDAT (www.eudat.eu).”
7. We removed a substantial part of the detailed description, including the background related to the project leader meetings. Also we added a short line on who initiates the topics for focus meetings (“These common interests can be signalled by the programme managers or brought up by DTL scientists”), but not in too much detail to keep this part short.
8. Yes, obviously it would be a two-way interaction, as is the basic idea of ELIXIR. To make this more clear we added: “Each of the nodes contributes specific expertise to the ELIXIR network. The Dutch node  contributes expertise related to interoperability, learning, and computer and network infrastructure. Through the network, DTL-associated scientists can benefit from all European contributions.”

Minor points: all adapted according to suggestion.
